# Cervical cancer: Challenges and prevention strategies: A narrative review

**DOI:** 10.1002/hsr2.2149

**Published:** 2024-05-30

**Authors:** Abate Wondesen Tsige, Dessale Abate Beyene

**Affiliations:** ^1^ Department of Pharmacy, Asrat Woldeyes Health Science Campus Debre Berhan University Debre Berhan Ethiopia

**Keywords:** cervical cancer, HPV vaccine, prevention, screening, treatment

## Abstract

**Background and Aims:**

Human papillomavirus (HPV) infections that continue to exist are the main cause of cervical cancer (CC), two‐thirds of CC occurrences worldwide are caused by HPV 16 and HPV 18, and 99.7% of CC tumors are linked to oncogenic HPV infection. To identify challenges of CC and its prevention and treatment modalities.

**Methods:**

This review examined the epidemiology, predisposing factors, genetic factors, clinical assessment methods, current treatment options, and prevention approaches for CC. We had perform a narrative data synthesis rather than a pooled analysis. A thorough literature search in pertinent databases related to CC was done with the inclusion of data that were published in the English language.

**Results:**

Early detection of CC is of utmost importance to detect precancerous lesions at an early stage. Therefore, all responsible agencies concerned with health should make all women aware of the benefits of CC screening and educate the general public. HPV vaccination coverage is very low in resource‐limited settings.

**Conclusion:**

To achieve the goal of eliminating CC as a public health problem in 2030, the World Health Organization will pay special attention to increasing HPV vaccination coverage throughout the world. To further improve HPV vaccine acceptability among parents and their children, safety‐related aspects of the HPV vaccine should be further investigated through post‐marketing surveillance and multicentre randomized clinical trials.

## 1 INTRODUCTION

Human papillomavirus (HPV) infections that continue to exist are the main cause of cervical cancer (CC), a malignant epithelial tumor that develops from healthy cervical epithelium through the progressive development of low‐grade and high‐grade intraepithelial lesions.^1^, ^2^, ^3^ The majority of people acquire HPV shortly after engaging in sexual activity, which means that it is most frequently transmitted to the vaginal canal.^4^, ^5^ The interaction of systemic and local cofactors, including HPV infection, which is a crucial component of CC development, promotes the malignant transformation of cervical cells.^6^ It is necessary for the emergence of low‐ and high‐grade squamous intraepithelial lesions (precancerous), which are the precursors to preinvasive cervical intraepithelial neoplasia (CIN grades 1, 2, and 3), and the progression to invasive CC.^4^, ^5^, ^7^ The likelihood that genital HPVs will function as carcinogens in the emergence of CC has been categorized based on the degree of the link with the disease. HPV types 6, 11, 42, 43, 44, 54, 61, 70, 72, and 81 are low‐risk types while types 16, 18, 31, 33, 35, 39, 45, 51, 52, 56, 58, 59, 66, 68, 73, and 82 are high‐risk or oncogenic types.^8^, ^9^ In addition, oral contraceptive use, sexual promiscuity, cigarette smoking, age, and parity have been associated with a high risk of CC.^10^, ^11^, ^12^, ^13^, ^14^, ^15^ CC is the fourth most common cancer in women worldwide due to inadequate screening in many regions of the world.^16^, ^17^

NHS England first aimed to tackle the burden of the disease by introducing a national cervical screening program in 1988, which has since seen a significant reduction in over a third of cases in England. CC screening is available from the age of 25, as the disease is rare among younger individuals.^18^

Africa has the highest regional incidence and fatality rates, with 7–10 times the rates of the Western world.^16^, ^19^ HPV vaccination, regular screening, and treatment of precancerous lesions can prevent the majority of instances of CC.^1^, ^20^ Because CC is a curable condition, tertiary interventions have helped to lower fatality rates.^21^ It is widely accepted that regular screening is the most important public health strategy for reducing the incidence of CC and its associated mortality. The ultimate goal of screening is to reduce the incidence and associated mortality from invasive CC.^22^ High levels of poverty, cultural issues, social justice, gender, race, ethnicity, and geography all play a role in the multifaceted effects of CC on communities. Compared to women in high‐income nations, poor women with advanced diseases have considerably higher mortality rates because they have less access to diagnosis and treatment.^23^, ^24^

## 2 EPIDEMIOLOGY OF CC

Globally, there has been a rise in the prevalence of CC among women under 40.^25^ The main reason for cancer‐related fatalities in women in Central America is CC.^26^ Geographical location has a significant impact on incidence and death. Since the implementation of official screening programs and HPV vaccination programs 30 years ago, the incidence and death of CC in high‐income nations have been reduced by more than half.^20^

CC is a serious issue in developing nations because of low public, medical professional, and policymaker awareness. Inadequate screening programs for precursor lesions and early‐stage cancer are also present, along with restricted access to healthcare services. Cancer is frequently discovered in later stages in women who have never had a screening, making treatment more challenging. Due to this, CC has a high incidence and fatality rate.^2^, ^27^ The majority of CC occurrences have been linked to HPV infections, with HPV 16 and HPV 18 being the most cancer‐causing subtypes, accounting for nearly 50% and 10% of cases, respectively.^26^ Sub‐Saharan African countries had the highest frequency of HPV infection among women with normal cytology (24%), followed by Eastern Europe (21%) and Latin America (16%).^28^ CC is still the most prevalent cancer in women and the leading cause of death in sub‐Saharan Africa, Melanesia, South America, and Southeast Asia despite primary protection from new vaccines and secondary prevention via cancer screening.^29^

## 3 ETIOLOGY OF CC

Clinical and epidemiologic evidence points to sustained oncogenic HPV infection as the main risk factor for the emergence of CC. Two‐thirds of CC occurrences worldwide are caused by HPV 16 and HPV 18, and 99.7% of CC tumors are linked to oncogenic HPV infection.^4^, ^30^ The risk of getting the HPV is highest in newly sexually active adolescent and young adult females.^31^ Shortly after sexual behavior begins, infection with HPV peaks.^4^ Nearly 90% of recently acquired HPV infections go undetected within 12–24 months,^31^ a condition that is sometimes referred to as “viral clearance” but which could potentially be immune control under the threshold of diagnosis or virus delay.^32^, ^33^ In approximately 60% of cases, there is a detectable immune response,^34^ as evidenced by the presence of serum antibodies specific to the type of HPV that caused the infection, and the ability of these antibodies to establish immunity to reinfection is uncertain.^35^ Low‐grade cervical cell alterations brought on by early HPV infection can be medically detectable. At 12–24 period of time they typically spontaneously revert. The risk of getting high‐grade cervical cell abnormalities, which can turn cancerous in 10–15 years if ignored, rises if infection with oncogenic HPV genotypes continues.^4^, ^36^

The study conducted by Bogani et al.^37^ reported that the incidence of recurrence was 7.46% for patients with HPV persistence at 6 months. The chance of having recurrent HPV infection is highly correlated with 12‐month HPV persistence (recurrence risk: 13.1%). However, a greater risk of recurrence was not correlated with HPV persistence lasting longer than 12 months.^38^

A significant factor in the emergence of invasive CC is the persistence of HPV infection, which is more prevalent in high‐risk or oncogenic forms.^39^

## 4 PREDISPOSING FACTORS OF CC

CC is predominantly caused by persistent HPV infections.^3^ It can be caused by HPV‐related factors like a young sexual onset, having several high‐risk intimate partners, and early age at first birth^40^, ^41^, ^42^ as well as non‐HPV‐related factors like poor socioeconomic status, contraceptive pills use, tobacco use, and genetics.^43^, ^44^, ^45^ The majority of people receive HPV right after beginning sexual activity, and this is the main way that it is spread. Additionally to direct sexual contact, skin‐to‐skin genital contact is another way that HPV infections can spread.^46^

### 4.1 Socio‐demographic and lifestyle‐related factors

Lower CC screening participation and awareness of HPV was one of the predicted factors for increasing the incidence of CC. In a study conducted in the United States of America, older female caregivers, racial/ethnic minorities, and individuals with less education had lower HPV awareness and CC screening adherence.^47^ Another investigation from Switzerland reported that lowered CC monitoring involvement was significantly strongly associated with overweight, lack of physical activity, and dietary inattention. likewise was also a statistically significant positive correlation between involvement in monitoring and having high cholesterol and a history of chronic disease.^48^ Additionally, a meta‐analysis indicated that there are ongoing disparities between social classes in the rate of CC. The reality that these variations are greater for cancer than for dysplasia indicates that behavioral factors, such as a female's partner's way of life, and her involvement in suitable screening programs, are significant predictors of these differences.^49^

### 4.2 Sexual activity

The most widespread infection transmitted through sexual contact in the world is the HPV, or HPV, which can infect up to 75% of sexually active people throughout their lifetime and infects 44% of people 24 months after first having sex.^5^, ^50^ Between 2 and 3 months of the initial sexual face, HPV infections were identified, and two‐thirds of the women were infected with the virus by the end of the 48‐month follow‐up period.^5^

### 4.3 Genetic factors

There is little known about the genetic variables that influence the onset of CC. Familial clustering, however, has been supported by data for more than 60 years.^51^ At the 6p21.3 locus of the human leukocyte antigens (HLA), several case‐control association studies have identified supporting evidence for several distinct risk variations. Recently published genome‐wide association research and the meta‐analyzes in vast populations have demonstrated CC susceptible variants that point to 2q14 (PAX8), 17q12 (GSDMB), and 5p15.33 (CLPTM1L) as reliably reproduced non‐HLA susceptibility loci.^52^ Overall four mutations in CTLA4 and HLA DQB1, moderate for the five variants in IL‐1B, IL‐10, XRCC3, and HLA DQA1, and weak for 10 variants, epidemiological evidence of relationship has been demonstrated.^53^

### 4.4 The use of oral contraceptive

In comparison to women who do not use contraceptive pills, women who have used contraceptive pills for more than 5 years are at a greater risk of getting CC.^54^ The International Agency for Research on Cancer (IARC), a division of the World Health Organization (WHO), reported that extended use of the oral contraceptive pill raised the risk of CC by up to four times.^55^ In one study, the risk was raised by 10% with fewer than 5 years of usage, by 60% with 5–9 years of use, and by twice with 10 or more years of use. However, it has been revealed that once women cease using contraceptive pills, their likelihood of developing CC substantially reduces.^45^, ^56^, ^57^ Following the suggestions for CC screening further decreases the elevated risk of CC brought on by these hormonal risk factors.^56^

### 4.5 Cigarette smoking

Smoking and the possibility of acquiring CC following a cervical HPV infection have been strongly linked by epidemiological studies.^58^, ^59^, ^60^, ^61^ In a thorough analysis of the available data, the IARC has also determined that smoking is a cause of CC.^62^ Even after accounting for HPV seropositivity, one study found that women who had given up smoking for a prolonged amount of time (20 years or longer) had a statistically significant twofold lower risk of illness than current smokers.^13^ According to the IARC pooled research, women who had given up smoking for less than 4 years remained to have a greater likelihood of getting cervical intraepithelial neoplasia grade 3 or higher (CIN3) in contrast to nonsmokers.^61^

According to certain research,^13^, ^63^ there is no connection between passive smoking and the incidence of CC. On the other hand, a meta‐analysis showed that passive smokers had a 2.8‐fold increased risk of CC compared with non‐smokers.^64^ This could be when assessing indirect smoking is challenging, and it might be especially challenging to account for residual confounding because the woman's male partner's smoking habits may be closely associated with his sexual activities and, thus, with the chance of transmission of HPV within the couple. If a risk factor for CC can be changed, quitting smoking effectively lowers the risk of the disease.

### 4.6 HIV infection

Squamous cell lesions (SIL), the precursor to cancer of the cervical cavity, and infection with HPV are more common in HIV‐infected women.^65^ Precancerous cervical lesions were more common, carcinogenic subtypes were more widespread, and lesions developed more quickly in women with low CD4 counts and high HIV viral loads.^66^, ^67^ These women also had a greater risk of persistent infection with several HPV subtypes. On the other hand, the risk of HPV infection was lower in women with anti‐retroviral treatment adherence and low CD4 count.^68^ Recurrent abnormalities are more common after treatment of precursor lesions, putting these women at a higher risk of dying from CC.^69^, ^70^ This method takes into consideration these relationships since they have significant effects on treatment regarding both HIV and CC.

## 5 SCREENING METHODS FOR CC

People with cervixes should regularly receive CC screenings, which are used to check for disease in the cervix before symptoms manifest.^27^ It is also used to detect precancerous lesions early so that they are more treatable and curable than advanced cancer.^71^ Only 12.87% of women in sub‐Saharan Africa receive CC screenings, which can be mainly explained by a systematic review and meta‐analysis.^72^

There are now three alternatives that are advised for CC screening for those between the ages of 21 and 65: primary HPV testing every 5 years, cytology alone every 3 years, and co‐testing with the combination of cytology and HPV testing every 5 years.^73^, ^74^ Primary HPV testing and co‐testing are more successful than cytology alone in identifying high‐grade cervical intraepithelial neoplasia in individuals aged 25–65 who are at moderate risk. Researchers in clinical trials, cohort research, and modeling investigations, however, indicated that colposcopy and the use of HPV‐based testing are linked with a higher risk of false‐positive results^75^, ^76^, ^77^ (Table 1).

**Table 1:** Current screening recommendations for cervical cancer.^75^, ^76^, ^77^, ^78^

Age in years	ACS (2012)	USPSTF (2018)	ACS (2020)
Age 21–24	3 years between Pap tests	3 years between Pap tests	Screening is not indicated.
Age 25‒29	3 years between Pap tests	3 years between Pap tests	Every 5 years is ideal for HPV testing. Every 5 years, an HPV/Pap test is acceptable. Every 3 years between Pap tests is acceptable.
Age 30‒65	Every 3 years HPV/Pap Co‐test is preferred. 3 years between Pap tests are acceptable.	Every 3 years is an ideal Pap test. There is either an HPV test or an HPV/Pap test performed every 5 years.	Every 5 years is ideal for HPV testing. An HPV/Pap test is permitted once every 5 years. It is acceptable to wait 3 years between Pap tests.
Age 65 and older	It is not advised to screen if several earlier tests came out normal.	You shouldn't get checked if a series of prior tests were negative and you weren't at a high risk for cervical cancer.	If several previous tests came up normal, no screening is required.

Abbreviations: ACS, The American Cancer Society; USPSTF, US Preventive Services Task Force.

Between the ages of 25 and 65, the American Cancer Society (ACS) suggests that people having the cervix have an HPV test as a component of a 5‐year CC screening program. We can use an HPV/Pap test every 5 years or a Pap test once every 3 years if HPV testing alone is not available. These recommendations differ slightly from those made by the US Preventive Services Task Force (USPSTF) in 2018 and the ACS in 2012^78^ (Table 1).

When compared to a Pap smear, a visual examination with acetic acid (VIA), or a cytology examination, the HPV DNA‐based test is considered by the most recent WHO guidelines as the most effective method to find cells that are developing cancer. Because it finds the high‐risk HPV strains that cause nearly all cases of CC,^79^, ^80^, ^81^ this test is utilized extensively throughout the world. It is a simpler and cost‐effective test that prevents more precancerous lesions and cancer and saves more lives.^82^, ^83^ To achieve the global strategy WHO goal of reaching a 70% testing rate by 2030, it is important to improve access to products and consider self‐collection of samples, as women often feel more comfortable taking their samples.^79^, ^84^

### 5.1 Screening methods of CC in special population

When it comes to pregnant mothers being screened for CC, it is recommended to delay routine recall and test of cure smear tests until 3 months after delivery.^85^ However, if a woman requires follow‐up after treatment for CC, cytology tests should not be delayed until after delivery. Colposcopy is a safe procedure during pregnancy and can be used to exclude invasive diseases. Biopsies should be deferred until after delivery, except in cases where invasive disease is suspected, and an adequate biopsy is necessary for proper diagnosis.^86^, ^87^

From 2017 to 2021, CDC's National Breast and Cervical Cancer Early Detection Program (NBCCEDP) provided timely breast and CC screening, diagnosis, and treatment to low‐income women with inadequate insurance.^88^ Many developing countries in Africa have not prioritized CC screening due to competing funding priorities low prioritization of CC and cultural practices across Africa.^89^, ^90^ The new guidelines from the ACS do not recommend Pap tests or HPV tests for individuals with a cervix who were exposed to diethylstilbestrol while in utero, those living with HIV, those who have undergone a total hysterectomy and no longer have a cervix, and those who have no history of CC or pre‐cancerous conditions.^71^

### 5.2 Cost‐effectiveness and implementation of CC screening

Regarding primary and secondary prevention of CC, WHO suggests that 90% of girls be fully vaccinated with HPV vaccines by age 15 and that 70% of women be screened with a high‐power test (preferably a test based on HPV DNA) by age 35, and again by age 45.^21^ A study conducted in China has found that implementing HPV‐based screening can be a cost‐effective approach, but only if routine vaccination rates reach 75% or higher.^91^ A systematic review of economic evaluations of CC screening methods showed that primary HPV DNA testing strategies are cost‐effective in several settings. Visual inspection with acetic acid may also be cost‐effective in certain settings, such as rural areas.^91^ Another study carried out in Sweden and Australia also showed that primary screening for HPV is better than both cytology‐based methods and primary HPV screening followed by cytology.^92^, ^93^ A study conducted in Nicaragua also has found that HPV testing is a more cost‐effective option than Pap testing. This is because HPV testing offers greater test sensitivity and requires fewer visits.^94^

## 6 COMPLICATIONS OF CC

If CC progresses to a later stage and affects other parts of the body, it can lead to complications. Symptoms such as bleeding, pelvic pain, and vaginal discharge are likely.^95^ The tumor may spread to different regions of the body, including the uterus, vagina, and pelvic wall.^96^ In some cases, the tumor can block one or both ureters, which can lead to kidney failure.^97^ When CC spreads to the bones, it can cause bone and back pain, and when it spreads to the lungs, coughing can occur.^98^, ^99^

## 7 SAFETY OF CC VACCINE

The HPV vaccination program has led to a significant decline in CC rates. However, in low‐income countries, where vaccination coverage is only 1%, the incidence of CC remains high.^100^ The 9‐valent HPV vaccine (Gardasil 9), the quadrivalent HPV vaccine (Gardasil), and the bivalent HPV vaccine (Cervarix) underwent several years of rigorous safety testing before being approved by the Food and Drug Administration.^101^ Clinical trials involving over 15,000 women and men for Gardasil 9, over 30,000 for Cervarix, and over 29,000 for Gardasil have demonstrated their safety.^102^

The HPV 6 and HPV 11, both of which are linked to genital warts and recurrent respiratory papillomatosis, as well as HPV 16/18, can be prevented by the Gardasil quadrivalent vaccine, created by Merck and Co., Inc. HPV 16/18, the two most dangerous types, are preventable with the Cevarix bivalent vaccine, made by GlaxoSmithKline Biologicals. With HPV 6/11/16/18/31/33/45/52/58 accounting for 90% of CC incidence globally, the Gardasil nonavalent vaccine can offer protection against this virus. The vaccine in question is the most recent and complete one on the market.^103^, ^104^, ^105^

Recent studies have revealed that more parents are expressing concerns about the safety of HPV vaccines, despite over 15 years of consistent evidence demonstrating their effectiveness and safety. A study from 2015 to 2018 showed a decline in the acceptance rate of HPV vaccines for children, from 13% to 23%.^106^ It is important to note that, like any vaccine or medicine, HPV vaccines can cause side effects such as pain, redness, swelling, dizziness, syncope (fainting), nausea, and headache. The CDC's Vaccine Adverse Event Reporting System (VAERS) reports these as the most common side effects. Furthermore, the rate of anaphylaxis following vaccination in the United States of America is reported to be 3 cases per 1 million administered doses.^102^ Another study was conducted in eight countries including Australia, Canada, Colombia, Denmark, Hong Kong, Mexico, Sweden, and the United States (including Puerto Rico) to evaluate the safety, tolerability, and immunogenicity of the 9‐valent HPV vaccine (9vHPV) in females aged 12–26 who had previously received the quadrivalent HPV (qHPV) vaccine. The results showed that administering a 3‐dose regimen of the 9vHPV vaccine to adolescent girls was highly effective in producing antibodies against HPV types 31/33/45/52/58, and generally well‐tolerated.^107^ The review study conducted in 2021 emphasized the need for more comprehensive, impartial, and long‐term studies to properly evaluate the safety and effectiveness of these vaccines for children. Additionally, the review pointed out that nearly all clinical trials on the safety of CC vaccines were funded by the vaccine manufacturers.^108^

## 8 CC PREVENTION AND CARE

### 8.1 Options for CC treatment

Healthcare providers should consider many factors when choosing treatment for CC. The cancer stage, general wellness status, preferences, fertility issues, goals of treatment, available choices, possible negative outcomes, and anticipated length of therapy are some of these.^109^ Treatment options for CC may include the following. Surgery, radiation therapy, chemotherapy, targeted therapy, and immunotherapy.^27^, ^110^ For treatment of CC, total vaginal hysterectomy, total abdominal hysterectomy, total laparoscopic hysterectomy, radical hysterectomy, modified radical hysterectomy, a radical trachelectomy, also called a radical cervicectomy, a bilateral salpingo‐oophorectomy, a total pelvic exenteration, artificial openings (stomas), and plastic surgery types of hysterectomy are used.^27^, ^111^ Radiation therapy that is administered externally as well as internally is used to treat CC individuals who have advanced stages of the disease. To improve the patient's quality of life and reduce their symptom burden, this form of therapy can also be utilized as palliative care^110^ (Table 2).

**Table 2:** Lists of cervical cancer treatments by stage of cervical cancer^1^, ^27^, ^108^, ^109^, ^110^, ^111^, ^112^, ^113^, ^114^, ^115^, ^116^, ^117^, ^118^, ^119^, ^120^

Cervical cancer stage	Characteristics	Indication of chemotherapy	Current recommended treatment
Stage 0/carcinoma in situ	Malignancy in its earliest stages that confines itself to your cervix's area.	No	Internal radiation therapy, conization, and hysterectomy.
Stage 1(A)	Just a magnifying glass can see the tumor, that remains microscopic.	Sometimes	Radical trachelectomy, total hysterectomy, removal of lymph nodes and modified radical hysterectomy, conization and internal radiation therapy
Stage 1(B)	The cancer continues to stay contained to the cervical cavity although it spreads further than 0.2 inches.	Usually	Radiation therapy with chemotherapy, chemotherapy and surgery, radical hysterectomy and removal of pelvic lymph nodes with or without radiation and chemotherapy, radical trachelectomy and radiation therapy
Stage 2(A)	Outside the cervical cavity and the womb, cancer has advanced, but, not into the parametria, the connective tissue level under it.	Yes	
Stage 2(B)	The cancer has grown across the parametria surrounding the cervical cavity and womb.	Yes	Internal radiation, radiation therapy combined with chemotherapy, surgical removal of lymph nodes, radiation therapy with or without chemotherapy, a clinical trial of chemotherapy combined with surgery, and a clinical trial of chemotherapy and radiation therapy followed by another chemotherapy are all examples of techniques that can be used to treat cancer.
Stage 3(A)	The pelvic wall was not influenced by malignancy, but it has grown to the bottom part of the genital area	Yes	
Stage 3(B)	The malignancy has become sufficiently large to impair either of the ureters, the malignancy has advanced to the pelvic wall, or it has led to either of the kidneys being enlarged or stopping functioning.	Yes	
Stage 4(A)	The cancer moved on from the pelvis to the bladder, rectum, and other areas.	Yes	
Stage 4(B)	The cancer travels to distant structures such as the pulmonary system, skeletons, or remote lymphatic systems.	Usually	Radiation therapy with or without chemotherapy

Chemotherapy drugs are administered in cycles followed by recovery periods. Most cycles last 1–3 weeks. After a recovery period, the cycle begins again. Chemotherapy doses may be given every few weeks, weekly, or several times a week, and chemo cycles vary depending on the drug given.^1^, ^112^, ^113^ Among the most frequently utilized chemotherapy medications to treat CC include cisplatin, carboplatin, gemcitabine, ifosfamide, irinotecan, paclitaxel, topotecan, and vinorelbine. The targeted treatments for CC comprise tisotumab vedotin and bevacizumab. Pembrolizumab immunotherapy has been suggested^1^, ^110^, ^111^, ^114^, ^115^, ^116^ for certain individuals whose cervical carcinoma carries the biomarker programmed death ligand 1 (PD‐L1). Adenocarcinoma of the cervix accounts for approximately 20%–25% of all cervical malignancies. PD‐L1 is more frequently expressed by squamous cell carcinoma than by adenocarcinoma. Diffuse PD‐L1 expression in squamous cell carcinoma patients is correlated with poor disease‐free survival and disease‐specific survival compared with marginal PD‐L1 expression, which is associated with a remarkably favorable prognosis. In adenocarcinoma, there is a survival benefit for patients with tumor lacking PD‐L1‐positive tumor‐associated macrophages.^117^

When chemotherapy is given along with radiation therapy, the common drugs are cisplatin or carboplatin given weekly through an IV line, and cisplatin and 5‐fluorouracil given every 3 weeks. If the cancer has spread or returned after treatment, treatment may include a combination of the following chemotherapy drugs: Cisplatin, carboplatin, paclitaxel, and topotecan^27^, ^118^ (Table 2).

The retrospective study of patients with CC, treated at the Royal Marsden Hospital, United Kingdom, between 2004 and 2014, 70% of women treated with systemic therapy for recurrent or metastatic CC subsequently received second‐line therapy with an ORR of 13.2%, a median PFS of 3.2 months and a median overall survival of 9.3 months.^119^

### 8.2 Prevention mechanism of CC

The epithelium of the lower genital tract can be examined through colposcopy, which is a low‐powered microscope that uses light for illumination. Colposcopy practice spread and evolved during the 1930s and 1940s in Europe, and it became more widely established through the expertize of individual practitioners.^120^ Colposcopy is a cornerstone of CC screening, prevention, and treatment.^121^ Colposcopy is a precise method to detect CIN and distinguish between high‐grade and low‐grade abnormalities. It can be used to inspect any epithelial surface in the lower genital area. Anyone requiring treatment must undergo a colposcopic evaluation.^122^

In the examination of abnormal or inconsistent CC screening tests, the National Library of Medicine, 2021 advises colposcopy.^120^ Colposcopy reveals precancerous lesions in the cervical region and enables preventative care of abnormalities that are unlikely to worsen.^121^ Cancerous lesions that are precancerous and abnormal CC screening tests are treated based on risk rather than outcomes, according to a research study carried out in the United States of America.^123^

The World Health Organization has suggested the following approaches to prevent CC: for the majority of women, only screening, triage, and treatment using HPV DNA as the primary screening test beginning at age 25 every 3–5 years. HPV DNA should be used as the primary screening test starting at age 30 and updated at intervals of 5–10 years for HIV‐positive women.^79^

#### 8.2.1 HPV vaccination

The spread of HPV infection and the occurrence of associated diseases can be protected by the quadrivalent vaccine Gardasil, the bivalent vaccine Cevarix, and the 9vHPV.^108^ Routine human HPV vaccination for girls and boys at the target age 11–12 years (but can be given as early as 9 years) as part of the American College of Obstetricians and Gynecologists recommended adolescent vaccination platform.^124^ After the initiation of HPV 16 and 18 vaccination, HPV infections decreased by 83% in girls aged 15–19 years and by 66% in women aged 20–24 years (up to 8 years).^125^ For girls aged 9–14 years, before they become sexually active, two doses 6 months apart are recommended. An interval of no more than 12–15 months is recommended to allow girls to complete the program on time before becoming sexually active.^126^

## 9 STRENGTH AND LIMITATION OF THE STUDY

The ability to utilize a thorough updated literature search in a pertinent databases related to CC were the strengths of this review. The primary limitation was the inclusion of only data that were published in the English language and this review did not include the finding of the study that may be unpublished.

## 10 CONCLUSIONS AND FUTURE PERSPECTIVES

There has been a rise in the prevalence of CC among women under 40 years in the world. Socio‐demographic and lifestyle‐related factors, sexual contact, genetic factors, use of hormonal contraceptives, cigarette smoking, and having HIV infection have linked to occurrence of CC.

Screening for CC is of paramount importance in detecting precancerous lesions early. Therefore, all responsible agencies concerned with health should make all women aware of the benefits of CC screening and educate society in general. HPV vaccination coverage is very low in resource‐limited settings. To achieve the goal of eliminating CC as a public health problem in 2030, the WHO plan will pay special attention to increasing HPV vaccination coverage throughout the world.

To further improve the acceptability of the HPV vaccine among parents and their children, safety‐related aspects of the HPV vaccine should be further investigated in post‐marketing surveillance and multicenter randomized clinical trials.

## AUTHOR CONTRIBUTIONS

**Abate Wondesen Tsige**: Conceptualization; investigation; funding acquisition; writing—original draft; validation; methodology; visualization; writing—review and editing; project administration; formal analysis; software; data curation; supervision; resources. **Dessale Abate Beyene**: Conceptualization; investigation; funding acquisition; writing—original draft; methodology; validation; writing—review and editing; visualization; software; formal analysis; project administration; data curation; supervision; resources.

## CONFLICT OF INTEREST STATEMENT

The authors declare no conflict of interest.

## TRANSPARENCY STATEMENT

The lead author Abate Wondesen Tsige affirms that this manuscript is an honest, accurate, and transparent account of the study being reported; that no important aspects of the study have been omitted; and that any discrepancies from the study as planned (and, if relevant, registered) have been explained.

## Data Availability

The data that support the findings of this study are available from the corresponding author upon reasonable request. The authors confirm that the data supporting the findings of this study are available within the article.
